# Depinning Transition of a Domain Wall in Ferromagnetic Films

**DOI:** 10.1038/srep14062

**Published:** 2015-09-14

**Authors:** Bin Xi, Meng-Bo Luo, Valerii M. Vinokur, Xiao Hu

**Affiliations:** 1 International Center for Materials Nanoarchitectonics (WPI-MANA), National Institute for Materials Science, Tsukuba 305-0044, Japan; 2 Department of Physics, Zhejiang University, Hangzhou 310027, China; 3 Materials Science Division, Argonne National Laboratory, Argonne, Illinois 60439, USA

## Abstract

We report first principle numerical study of domain wall (DW) depinning in two-dimensional magnetic film, which is modeled by 2D random-field Ising system with the dipole-dipole interaction. We observe nonconventional activation-type motion of DW and reveal the fractal structure of DW near the depinning transition. We determine scaling functions describing critical dynamics near the transition and obtain universal exponents establishing connection between thermal softening of pinning potential and critical dynamics. We observe that tuning the strength of the dipole-dipole interaction switches DW dynamics between two different universality classes, corresponding to two distinct dynamic regimes characterized by non-Arrhenius and conventional Arrhenius-type DW motions.

Motion of domain walls in magnetic nanowires and films is a key component of operation of any magnetic memory and logic device^1^,^2^,^3^. To a great extent DW dynamics is governed by pinning-depinning processes which control the operational speed and power consumption of a device and thus play central role in device performance^4^,^5^. There has been remarkable progress in description of pinned DW dynamics based mostly on the elastic manifold model in a random environment^6^. A key property of such a system is that at zero temperature it experiences the *dynamic* phase transition (depinning transition): At small external drives, *F* ≤ *F*_*c*_, where *F*_*c*_ is the critical pinning force, DW is immobilized (pinned) by disorder, whereas at *F* > *F*_*c*_ it acquires a finite velocity *v*. The threshold depinning force *F*_*c*_ is a critical point in a sense that at 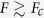, the velocity exhibits critical behavior *v* ~ (*F* − *F*_*c*_)^*β*^, as was proposed by Fisher^7^ in the context of depinning of charge density waves. At finite temperatures the velocity is always finite, and at *F* ≪ *F*_*c*_ the DW exhibits highly nonlinear glassy response, so-called *creep* dynamics, with 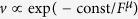
6. The depinning transition gets rounded and acquires a meaning of the intermediate region separating the low force creep dynamics from the asymptotic linear response *v* ∝ *F* at *F* ≫ *F*_*c*_. The critical depinning behavior has to include temperature dependence and was conjectured to be of the form: *v* ~ Ψ[(*F*/*F*_*c*_ − 1)/*T*^*η*^]^8^,^9^.

The above results were obtained in the framework of the elastic manifold model for DW, in which DW is viewed as an elastic membrane in 3D magnets and as an elastic string in 2D magnetic systems. Many of them were verified by computer simulations based on the Langevin dynamics of the elastic manifold^10^,^11^,^12^,^13^. However, despite many impressive successes, this description misses important processes that may become essential for the DW dynamics at elevated temperatures, in particular that the DW can become multi-valued, so that the ahead boundaries can merge with the ‘main’ part of the interface that moves behind.

This poses a challenge of developing a first principle approach starting from the microscopic model that captures basic physics of the magnetic system. Taking up upon this challenge we reveal the fractal structure of the DW and uncover the critical dynamics at the depinning transition. Furthermore, we uncover the role of strength of the dipole-dipole interaction in determining the proper dynamic universality class.

## Results

### Model

We model the two-dimensional (2D) magnet subject to quenched disorder by the 2D random-field Ising model with the dipole-dipole interaction, and the dynamics of which is controlled by the external driving magnetic field *H*. The Hamiltonian of the system is


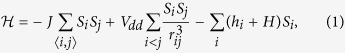


with *S*_*i*_ = ±1 at site *i*. The first term of the Hamiltonian is the ferromagnetic coupling between one spin and its nearest neighbors. Hereafter we measure the energy in the units of the coupling *J* and the distance in the units of elemental spin spacing. The second term is the magnetic dipole-dipole interaction with *r*_*ij*_ = |*i* − *j*| and *V*_*dd*_ a parameter for interaction strength. The on-site random field *h*_*i*_ distributes uniformly within an interval [−Δ, Δ] which generates random pinning potentials.

### Zero-temperature depinning

To come up with the quantitative description of depinning, we have to know its key characteristic, the zero-temperature depinning field *H*_*c*_. Finding its true value is a challenge since in finite systems realizations of the random potential fluctuate, and so do the corresponding values of the depinning field.

**Figure 1:**
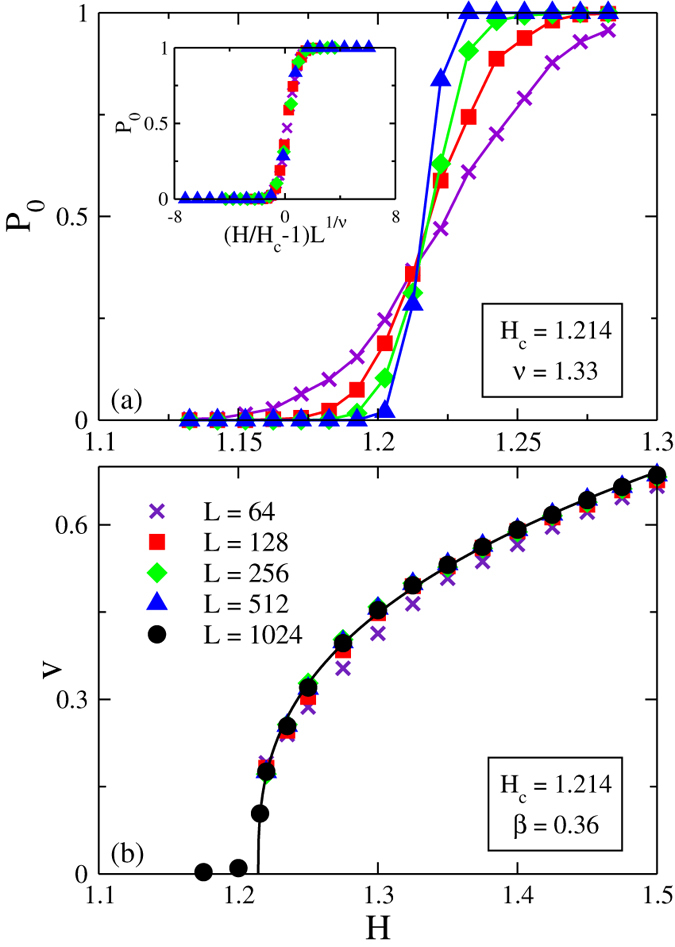
(**a**) The probability *P*_0_ versus the driving field with different system sizes and the corresponding scaling plot (inset). *V*_*dd*_/*J* = 0.1 and Δ = 1.5 *J* are used all through this work. (**b**) *v* − *H* characteristics at zero temperature with different system sizes. The solid curve is the fitting with function Eq. (3) to data for *L* = 1024.

It is observed that for a given field and system size, the DW may either be pinned inside the sample, or it may go through from edge to edge. We call the latter case a depinning event and evaluate the corresponding depinning probability *P*_0_. To determine a true value of *H*_*c*_, one thus has to perform the finite-size scaling analysis of *P*_0_ which would contain *H*_*c*_ as a parameter. As shown in Fig. 1a, *P*_0_(*L*, *H*) increases sharply as function of the magnetic field in the interval *H* = 1.1 ± 1.3. The curves corresponding to different system sizes cross at point of *P*_0_ = 0.38 ± 0.04 at *H* = 1.214 ± 0.006. This determines the depinning field which does not depend on the system size and thus can be taken as a depinning field *H*_*c*_ of a macroscopic system.

The problem of depinning at zero temperature is intimately related to percolation problem^14^. We thus assume that the depinning probability function has the form characteristic to the percolation problem^15^:


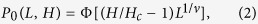


where *ν* is a universal exponent. By choosing the variable (*H*/*H*_*c*_ − 1)*L*^1/*ν*^ with *ν* as an adjustment parameter, we find that at *ν* = 1.33 ± 0.05, all the data points of *P*_0_(*L*, *H*) collapse onto a single curve as shown in the inset of Fig. 1a. This procedure defines the exponent *ν*.

Now we are equipped to study the *v*-*H* characteristics in the depinning regime. We start with the zero-temperature behavior. The results for *v*-*H* for systems of different sizes are displayed in Fig. 1b. One sees that for *L* ≥ 128 the curves do not practically depend on the size of the system. Assuming the standard *v*-*H* depinning relation^16^


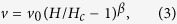


where *β* is a universal exponent, one finds *v*_0_ = 1.163 ± 0.005, *H*_*c*_ = 1.214 ± 0.006 and *β* = 0.36 ± 0.01 for *L* = 1024. This value is in a fair agreement with the *β* = 0.31 result obtained in two-loops RG calculations^17^ showing that the elastic manifold approximation works pretty well at zero temperature.

### Finite-temperature depinning

**Figure 2:**
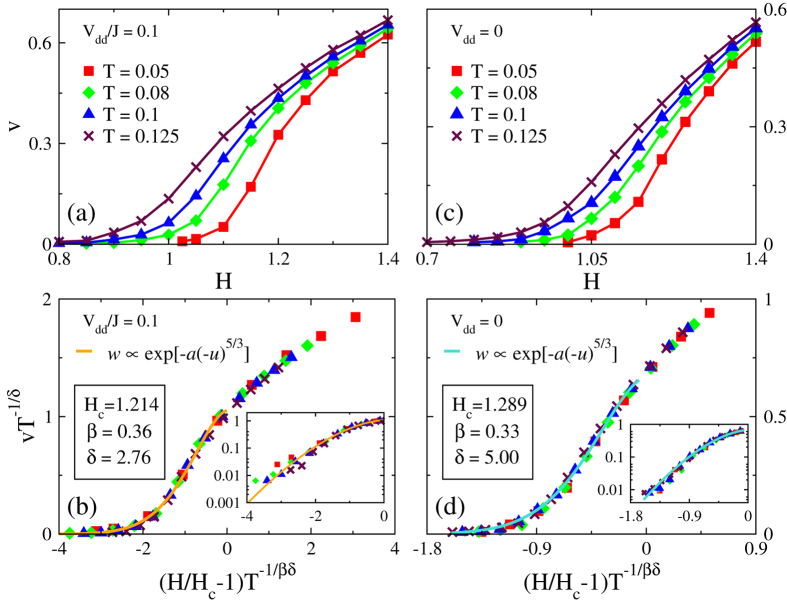
(**a**) Finite temperature *v* − *H* characteristics for *V*_*dd*_/*J* = 0.1. (**b**) Scaling plot of *v* − *H* curves as *vT*^−1/*δ*^ vs. (*H*/*H*_*c*_ − 1)*T*^−1/*βδ*^. The inset shows the same scaling behavior replotted in the semi-log scale. (**c**) Finite temperature *v* − *H* characteristics for *V*_*dd*_ = 0. (**d**) The corresponding scaling plot for *V*_*dd*_ = 0, the inset shows the same data in semi-log scale.

Now we turn to our main task, the finite temperature motion. To reduce the computation time we choose *L* = 512 system. Figure 2a shows the expected increase in velocity at the given field upon increasing temperature and an appreciable tail below the depinning field due to thermally activation processes.

We use the standard scaling ansatz^18^,^19^,^20^:


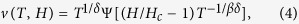


with Ψ(*x*) ~ *x*^*β*^ as *x* → ∞. We achieve the best collapse of the data to a single curve with *δ* = 2.76 ± 0.02 (which gives *βδ* = 1.00 ± 0.03) by adopting the values of *H*_*c*_ and *β* determined above, see Fig. 2b. Note, that these results cease to hold for large dipole-dipole interaction, *V*_*dd*_/*J* > 0.5 where the ferromagnetic order is broken.

At *x* < 0 the scaling function exhibits the asymptotic behavior 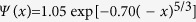 with error bars 5/3 ± 0.26, see Fig. 2b, and one arrives at the dynamics of the DW across the transition^20^ given by:


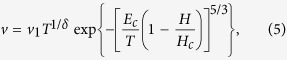


where *E*_*c*_ ≈ 0.81 is an energy barrier which governs the DW velocity at finite temperatures, and the condition *βδ* = 1 is taken into account. Note that the DW motion is not the conventional Arrhenius type. While a rigorous derivation of the temperature behavior of the velocity in the critical region is beyond the scope of the present work and will become a subject of forthcoming publication, we would like to call attention to the coincidence of the temperature dependence of Eq. (5) with that for the *creep* of the DW at *H* ≪ *H*_c_^6^, where the creep velocity is 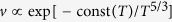 as the result of thermal softening of pinning potential^6^. Juxtaposing the *v*-*H* curves for *V*_dd_ ≠ 0 with those for *V*_*dd*_ = 0 shown in Fig. 2b, one observes that in the latter case the scaling treatment yields 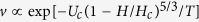 with error bars 5/3 ± 0.17, see Fig. 2d, i.e. the Arrhenius activation behaviour with the barrier that scales as *U*_*c*_(1 − *H*/*H*_*c*_)^5/3^, where *U*_*c*_ ≈ 0.67 is the bare energy barrier with *H*_*c*_ = 1.289 ± 0.002, *β* = 0.33 ± 0.01 and *δ* = 5.00 ± 0.01 (which gives *βδ* = 5/3 ± 0.05).

**Figure 3:**
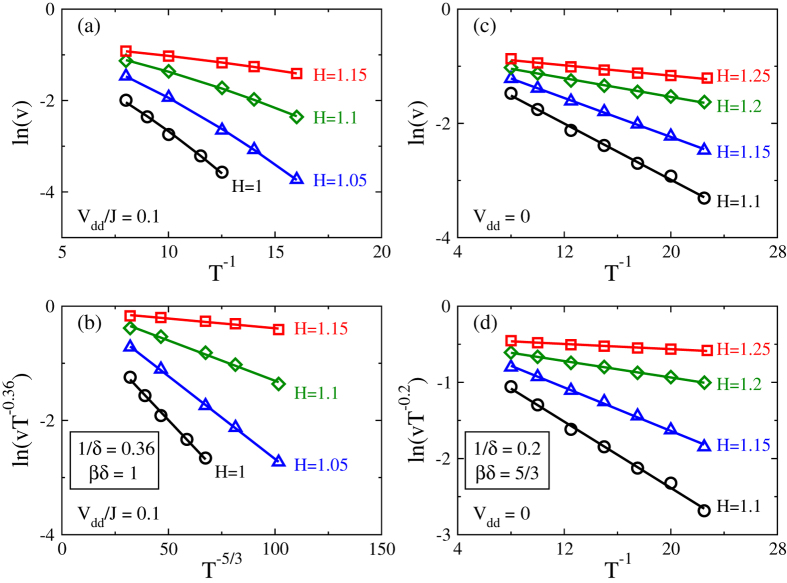
(**a**) Semi-log plot for velocity versus inversed temperature for the case with dipole-dipole interaction. (**b**) Semi-log plot for data in (**a**) taking into account the critical exponents evaluated in Fig. 2b. (**c**) Semi-log plot for velocity versus inversed temperature for the case without dipole-dipole interaction. (**d**) Semi-log plot for data in (**c**) taking into account the critical exponents evaluated in Fig. 2d. The straight lines in (**b**,**d**) indicate a common scaling function 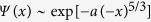 with *x* = (*H*/*H*_*c*_ − 1)*T*^−1/*βδ*^.

In order to cross-check our results and to better illustrate the effect of the dipole-dipole interaction on the temperature dependence of thermally-activated DW motion, we present the velocity *v* as a semi-log plot vs. the inverse temperature *T*^−1^ for representative magnetic fields below the critical depinning field. As shown in Fig. 3a, curves are convex provided the dipole-dipole interaction is switched on. This means that *v*(*T*) is a compressed exponential function with the temperature power larger than unity implying a non-Arrhenius-type behavior. Note that Fig. 3c showing the results without the dipole-dipole interaction displays the conventional Arrhenius-type DW motion. Furthermore, one can make semi-log plot for velocity vs. temperature with the power determined by critical exponents evaluated via the scaling analysis. As displayed in Fig. 3b,d, now straight lines are obtained for both cases meaning pure exponential functions with arguments of temperature to the right powers. This offers an unambiguous crosscheck for the non-Arrhenius-type motion in the presence of dipole-dipole interaction. The behaviors summarized in Figs 2 and 3 constitute the main results of our work.

Note that at finite temperatures the definition of the depinning field *H*_*c*_ is not straight forward. Our approach offers a systematic way for analyzing data at finite temperatures yielding *H*_*c*_ and the thermal activation energy barrier *E*_*c*_ simultaneously. Importantly, *E*_*c*_ depends not only on the strength of randomness Δ but also on the competition of the exchange coupling *J* and the dipole-dipole interaction strength *V*_*dd*_.

### Domain-wall morphology

**Figure 4:**
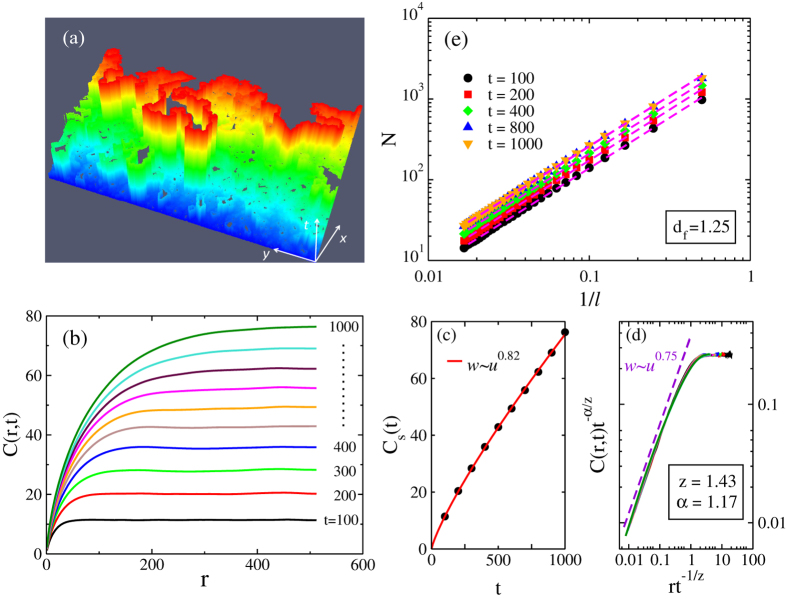
(**a**) Time evolution of DW near the depinning threshold at zero temperature. (**b**) Height-difference correlation function *C*(*r*, *t*) versus *r* for different values of *t* near the depinning threshold. (**c**) Saturated value *C*_*s*_(*t*) versus *t*. (**d**) Scaling plot for data in (**b**) with the dashed line 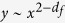. (**e**) Log-log plot of *N* versus 1/*l* at different times, with *l* the ruler size and *N* the measured length in units of *l*. Parameters used in calculations are: *L* = 1024, *V*_*dd*_/*J* = 0.1 and *H* = 1.22.

Next we investigate the DW morphology during the depinning process for the system with *V*_*dd*_/*J* = 0.1. To this end we set a flat DW along *y* axis at *x* = 1 at *t* = 0 with *H* = 1.22 just above *H*_*c*_ = 1.214, and drive it along *x* direction at zero temperature. As shown in Fig. 4a, the DW evolves rougher with time, and develops a fractal structure. Moreover, there remain several small unflipped-spin areas (black puddles) forming the “lakes” inside the domain. The multiconnected nature of the flipped domain originates from spatial fluctuations of the depinning field due to random character of the pinning potential: there are lacoons where the local depinning field still exceeds the driving field. The “overhangs” of the frontier of the DW are of the same origin. To quantify this multi-valued DW morphology, we define the modified height function *h*(*y*, *t*)^21^


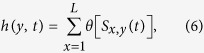


with *θ*(*x*) the unit step function and *S*_*x*,*y*_(*t*) the spin value on the site (x, y) at time *t*. The function *h*(*y*, *t*) describes the total number of flipped spins along the line *y* at time *t*. It is obvious that *h*(*y*, *t*) describes the position of DW if there is no “lakes” and “overhangs”.

The height-difference correlation function *C*(*r*, *t*) describes the DW roughness characteristics and is define as 21,22,23:


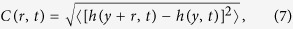


with *r* the distance between two points in *y* direction.

As shown in Fig. 4b, for fixed *t*, *C*(*r*) increases from zero with *r* and saturates at large *r*, and the saturated value *C*_*s*_(*t*) increases with *t*. These properties can be understood from the time-evolution process of DW morphology as displayed in Fig. 4a. The initial DW is a straight blue line with no height difference. By applying driving field, locally meandering segments appear first (see the light blue regions in Fig. 4a). Correlation of DW positions only exists in a small length scale. As time evolves, the meandering segments spread out along both the DW 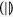 and moving (┴) directions, leading to rougher structures. There are two *t*-dependent correlation lengths: 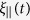 and *ξ*_┴_(*t*), growing with time as 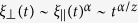 with *z* the dynamic exponent and *α* the roughness exponent, and the correlation function evolves as^21^:


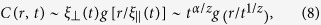


with *g*(*x*) saturates at constant as *x* >> 1.

As displayed in Fig. 4c, we obtain *α*/*z* = 0.82 ± 0.01 in terms of *C*_*s*_(*t*) ~ *t*^*α*/*z*^ in the large *r* limit of Eq. (8). By choosing *z* = 1.43 ± 0.01, all the data collapse into a single curve as displayed in Fig. 4d, which determines the dynamics exponent *z*. The roughness exponent *α* is then estimated as *α* = 1.17 ± 0.02. For comparison, we obtain *α* = 1.27 ± 0.02 and *z* = 1.52 ± 0.01 for the case without dipole-dipole interaction (raw data not shown explicitly in the present work). For the short-ranged elastic line model, *α* = 1.26 ± 0.01 was obtained in^11^, and *α* = 1.25 ± 0.05 and *z* = 1.42 ± 0.04 were obtained in^24^. The exponents *α* and *z* are not very different in the two cases with and without dipole-dipole interactions. The values (without dipole-dpiole interaction) are also close to those of short-ranged elastic line model.

We then study the fractal geometry of the DW near the depinning region. The fractal structure shown in Fig. 4a does not have a counterpart in the elastic line model, since a fractal-nature curve is not differentiable, which is required for applicability of the elasticity theory for the interface dynamics. For a fractal structure, the measured length *N* in units of ruler size is related to the ruler size *l* by: 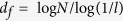, with *d*_*f*_ the fractal index. Through log-log plot of *N* versus 1/*l* as shown in Fig. 4e, we obtain *d*_*f*_ = 1.25 ± 0.01 (*d*_*f*_ = 1.20 ± 0.01 for the case without dipole-dipole interaction, raw data not shown explicitly in the present work). We notice that 
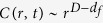
 only holds for 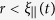 21. As shown by the dashed line in Fig. 4d, the exponent 2 − *d*_*f*_ ≈ 0.75 appears in the small scaling variable *r*/*t*^1/*z*^ limit of function *g*(*r*/*t*^1/*z*^). It is noticed that the fractal index is related to the local interface fluctuations through the so-called Hurst exponent *H* = *D* − *d*_*f*_ with *D* being the space dimension^25^,^26^, while the roughness exponent *α* describes the global interface fluctuations.

## Discussion

Numerical studies of depinning of the domain wall have been carried out mostly in the framework of the Langevin dynamics of the elastic line, see, for example^10^,^11^,^12^,^13^. There have been also first principle Monte Carlo (MC) simulations of the DW dynamics in the framework of the random-field Ising model^18^ in the absence of the dipole-dipole interaction, which also revealed scaling behavior. We employed the first principle MC calculations to explore depinning dynamics of magnetic DW with the tunable dipole-dipole interaction included into the random-field Ising model studied in^18^. We found that the critical exponents in presence of the dipole-dipole interaction differ from those without interaction. Our results indicate that switching on dipole-dipole interaction changes the universality class of the system. Note that both an analytical work on long-range-correlated disorder^27^ and the numerical study of the long-ranged elasticity^28^ also suggested the existence of different universality classes as compared with the short-ranged ones, although different from those of the present case dominated by dipole-dipole interaction. The existence of two depinning universal classes in elastic manifolds in random pinning potentials has been addressed by two of the present authors^20^ in the context of vortex dynamics in type-II superconductors, where Bragg glass and amorphous vortex glass (AVG) correspond to weak and strong random pinning potentials. Heavily disordered amorphous vortex glass (AVG) phase demonstrated the Arrhenius-type depinning dynamics, whereas Bragg glass state exhibited non-Arrhenius-type behavior. The DW system with dipole-dipole interaction is another example which exhibits non-Arrhenius-type dynamics at depinning fixed point. In simple physical terms, the non-Arrhenius behavior in the present work can be thought as the result of the enhanced flexibility of DW by dipole-dipole interaction, which favors interactions with the defects in the immediate vicinity of DW and the DW becomes more sensitive to thermal-fluctuation effects. The intriguing relation of this reasoning with the observed change in the universality upon tuning the dipole-dipole interaction calls for further research.

## Methods

Our simulations are performed on *L* × *L* square lattice. A flat DW between spin +1 and spin −1 is created along *y* axis at *x* = 1 as the initial condition. The magnetic field is applied to drive the DW in the positive direction of *x* axis in accordance to the Metropolis algorithm with single-spin flip^29^. Periodic boundary condition (PBC) is adopted at the DW (*y*) direction, whereas Anti-periodic boundary condition (APBC)^18^ at the moving (*x*) direction. The number of independent runs is at least 3000. The time unit is defined by a sweep of MC trials over the whole system, and the velocity is defined by *v* = *dM*/2*L dt* in steady states, with *M* the total magnetization. For a small system under large driving field, it reaches a steady state quickly (*t* ~ 10 for warm-up and ~10^2^ for statistics), whereas typically 10^3^ time steps for warm-up and statistics with regard to large systems under critical driving field. The time scale in MC technique should be proportional to the real time, but a straightforward relation is not easy. In order to derive the correspondence, one need to compare the simulation results and experiments at least once.

The system adopted in the present work is a coarse-grained one. The on-site spin is represented in terms of a block spin which contains *n*_*z*_ × *n*_*xy*_ × *n*_*xy*_ unit cells in a thin magnetic film, with *n*_*z*_ and *n*_*xy*_ the number of unit cells along the out-of-plane and in-plane direction, respectively. Then the energy unit *E*_*J*_ = *n*_*z*_*n*_*xy*_*aA*, with *a* the lattice constant of a real material and *A* the corresponding exchange stiffness. For *Nd*_2_*Fe*_14_*B*, *a* = 0.88 nm and *A* = 7.7 pJ/m^30^, whereas *a* = 0.25 nm and *A* = 10.3 pJ/m for Co layer in Pt/Co/Pt thin films^30^,^31^. The real temperature can be given in terms of the dimensionless temperature *T* in the present work by *T* ⋅ *E*_*J*_/*k*_*B*_, with *k*_*B*_ the Boltzmann constant. Taking *n*_*z*_ = *n*_*xy*_ = 1, one can have *T* = 0.1 approximates to 49 K for Nd_2_Fe_14_B, whereas 19 K for Pt/Co/Pt thin film.

## Additional Information

**How to cite this article**: Xi, B. *et al.* Depinning Transition of a Domain Wall in Ferromagnetic Films. *Sci. Rep.* **5**, 14062; doi: 10.1038/srep14062 (2015).
